# Using King Vision video laryngoscope with a channeled blade prolongs time for tracheal intubation in different training levels, compared to non-channeled blade

**DOI:** 10.1371/journal.pone.0183382

**Published:** 2017-08-31

**Authors:** Marc Kriege, Christian Alflen, Ruediger R. Noppens

**Affiliations:** 1 Department of Anesthesiology, University Medical Center of the Johannes Gutenberg-University, Mainz, Germany; 2 Department of Anesthesia & Perioperative Medicine, Western University, London, Ontario, Canada; University of Queensland, AUSTRALIA

## Abstract

**Purpose:**

It is generally accepted that using a video laryngoscope is associated with an improved visualization of the glottis. However, correctly placing the endotracheal tube might be challenging. Channeled video laryngoscopic blades have an endotracheal tube already pre-loaded, allowing to advance the tube once the glottis is visualized. We hypothesized that use of a channel blade with pre-loaded endotracheal tube results in a faster intubation, compared to a curved Macintosh blade video laryngoscope.

**Methods:**

After ethical approval and informed consent, patients were randomized to receive endotracheal Intubation with either the King Vision^®^ video laryngoscope with curved blade (control) or channeled blade (channeled). Success rate, evaluation of the glottis view (percentage of glottic opening (POGO), Cormack&Lehane (C&L)) and intubating time were evaluated.

**Results:**

Over a two-month period, a total of 46 patients (control n = 23; channeled n = 23) were examined. The first attempt success rates were comparable between groups (control 100% (23/23) vs. channeled 96% (22/23); *p* = 0.31). Overall intubation time was significantly shorter with control (median 40 sec; IQR [24–58]), compared to channeled (59 sec [40–74]; *p* = 0.03). There were no differences in glottis visualization between groups.

**Conclusion:**

Compared with the King Vision channeled blade, time for tracheal intubation was shorter with the control group using a non-channeled blade. First attempt success and visualization of the glottis were comparable. These data do not support the hypothesis that a channeled blade is superior to a curved video laryngoscopic blade without tube guidance.

**Trial registration:**

ClinicalTrials.gov NCT02344030

## Introduction

It is generally accepted that direct laryngoscopy using a Macintosh blade is a difficult skill to master [1]. The limitations of direct laryngoscopy are well known. A minimum number of 50 intubations is necessary to achieve a first pass Intubation success rate of >85% using direct laryngoscopy [2–4]. To obtain optimal visualisation of the glottis, direct laryngoscopy requires alignment of the oropharyngeal-laryngeal axes. However, duration of intubation and the success rate for securing the airway by tracheal intubation might have a significant impact on undesirable events like hypoxia or regurgitation.

Recently, the use of video laryngoscopy has become a widely accepted method in both emergency medicine and clinical anesthesia. It facilitates easy visualization of the glottis without a direct line of sight [5–6]. Ease of handling, high success rate in patients with normal and with difficult airways, high success rate in difficult airway situations and a steep learning curve makes these devices very popular among physicians [7–8].

It can sometimes be challenging to place an endotracheal tube (ETT) in front of the glottis and advance it despite good visualization on the monitor, especially when a video laryngoscope (VL) with a hyper-angulated blade is used [9]. This phenomena (great view but unable to intubate) is linked to VL blades that are, unlike the traditional Macintosh blade, hyper-angulated. Because of the unique profile that follows the anatomical shape of the human airway, alignment of the oropharyngeal-laryngeal axes becomes unnecessary to visualize the glottis. The new challenge is now to also bring the tip of the ETT to the level of the glottis, pass the glottis and advance the tube inside the trachea. Several techniques have been proposed to meet this challenge; many authors proposed using a stylet to give the tube the shape of a “hockey stick” to follow the curvature of the video blade [10]. However, ETT placement is often associated with a prolonged time for intubation [11]. Additionally, stylet use for video laryngoscopy has been linked to an increased risk of soft tissue injury of the upper airway [12]. VLs with an embedded tube-guiding channel might resolve this issue, because the tube is already loaded on the curved blade allowing the tube to be advanced once the glottis is visualized. This technique removes the need for a stylet and additional manipulations to steer the tube through the upper airway.

**Fig 1:**
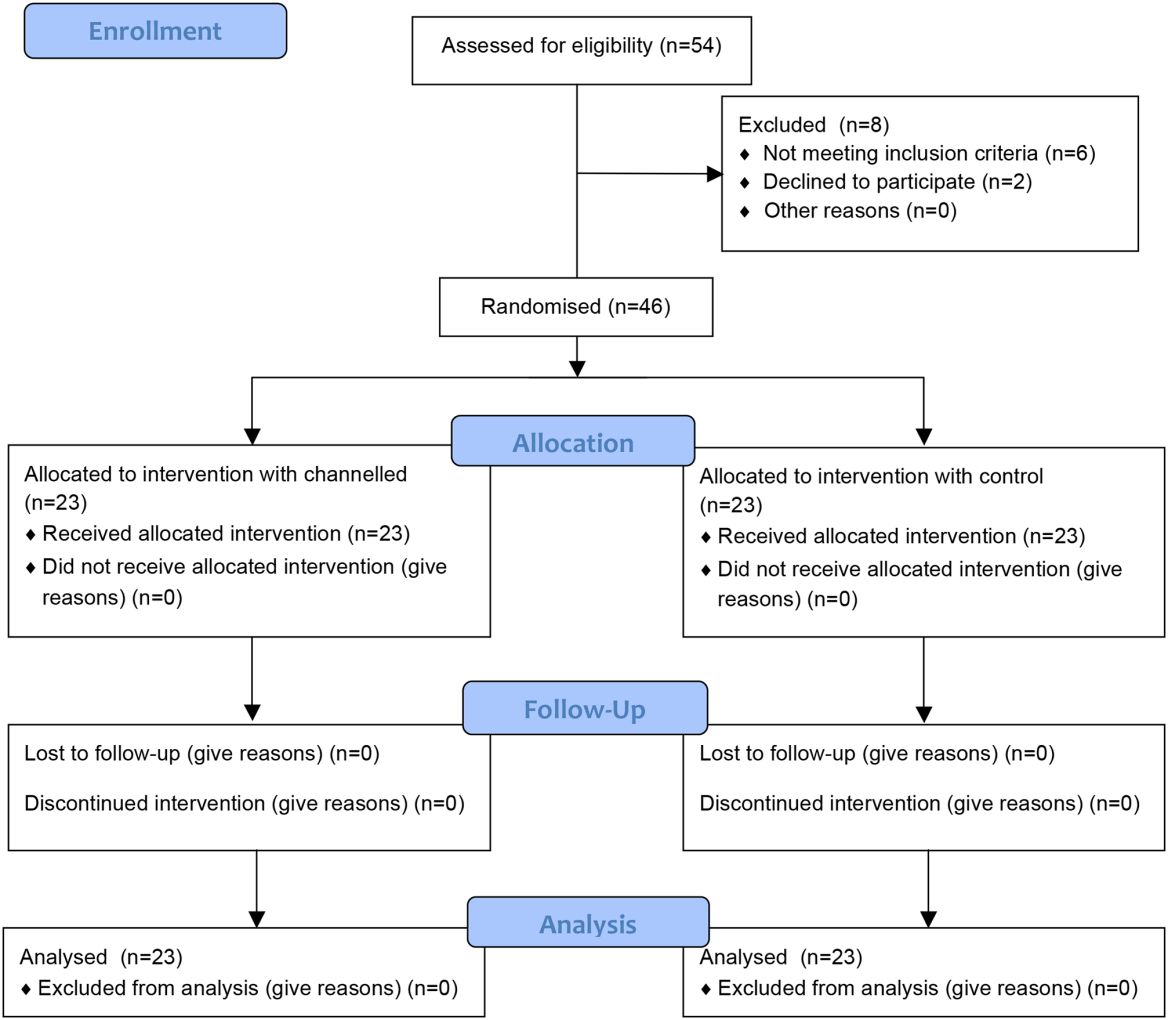
CONSORT flow diagram.

We hypothesized that the use of a VL with a curved, channeled blade allows faster intubation compared to a similar-shaped video blade without a guide channel (Fig 1). The King Vision VL was used for this trial because both blade types with a similar shape are available for this device. The only difference between these single-use blades is the presence of tube guidance in the channeled blade. The term “tube-guiding” is representative for the King Vision channeled blade in this manuscript.

## Methods

The ethics committee of the Medical Association of the state of Rhineland Palatine (Germany) approved this trial (Registration Nr.: 837.403.14 (9642)). This study is registered with ClinicalTrial.gov register number NCT 02344030. Written informed consent was obtained from all patients at least 18 hours before randomization.

After sample size calculation, a prospective, randomized, controlled trial (S1 File) in patients undergoing general anesthesia at a tertiary university hospital (Anesthesia Division of the Departments of Ear-nose-throat Surgery and Ophthalmic Surgery) was designed to examine the hypothesis that a video laryngoscope with a guide channel is more superior (S2 File).

**Fig 2:**
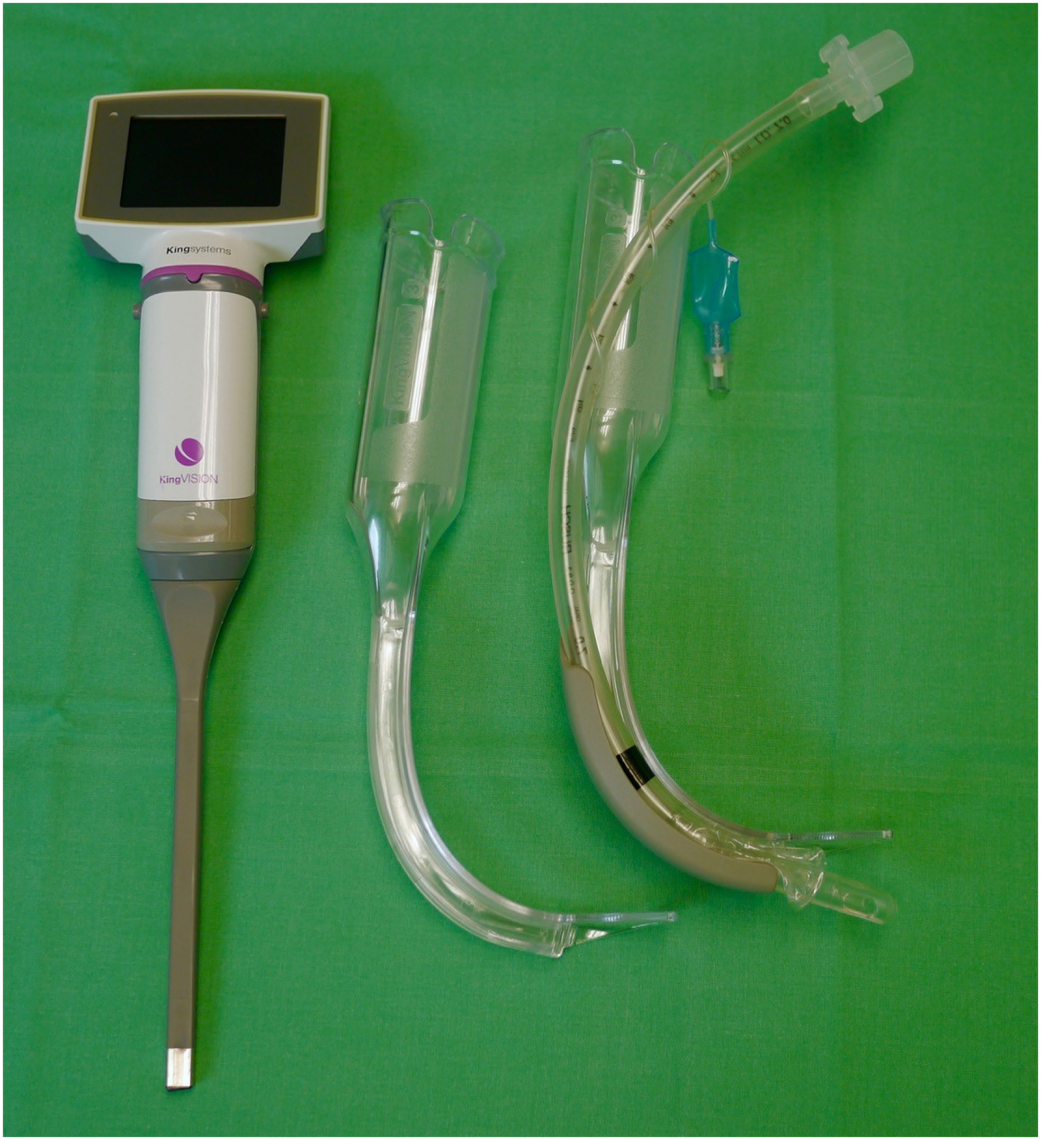
King Vision with non-channeled and channeled blades.

The King Vision^™^ aBlade VL (KV; Ambu GmbH, Bad Nauheim, Germany) was used for this study. For the KV, two types of blades are available: one with an integrated tube guiding channel (channeled) and a non-channeled version (control; Fig 2). Both models have high blade angulation (blade angle 90 degrees) with a similar shape; the only difference is the presence of a tube guide channel. The KV device consists of a 2.4-inch reusable OLED-Display (organic light emitting diode) and a disposable rigid blade that also includes a CMOS (Complementary metal-oxide-semiconductor) video camera (Fig 2). The KV is a wireless and fully portable VL with a high blade angulation, making direct visualization of the glottis unlikely. The blade size #3 is provided for adult use (tube sizes 6.0 to 8.0 mm). In total, 25 anesthesiologists participated in this trial; all were previously trained with video laryngoscopes and had sufficient clinical experiences using the device (Table 1). Each physician was given a standardized demonstration of the King Vision by one of the investigators: after an introduction which included handling as well the specifics of the device an intubation procedure was demonstrated.

**Table 1:** Data collection.

Patient characteristics	Control (n = 23)	Channeled (n = 23)	*p*-value
**Age** (years)	44 (23–89 [31–64])	48 (20–72 [32–58])	0.79
**Gender** (female/male)	12/11	11/12	0.76
**BMI** kg.m^2^	25.3 (21–32 [23.8–26.8])	26 (20–34 [24.1–30.5])	0.32
**ASA** class	2 (1–3 [1–3])	2 (1–3 [1–3])	0.54
**Provider** (n = 25)			< 0.001
Residents (n = 18)	16/23 (69%)	18/23 (78%)	0.37
Specialists (n = 7)	7/23 (31%)	5/23 (22%)	1.0
**Anesthesia experience** (months)	15 (5–200 [6–96])	23 (2–180 [12–72])	0.82
**VL experience** (n = 25) (applications)			0.87
**<10**	1	0	
**>10**	6	6	
**>25**	6	7	
**>50**	10	10	

Data are given as Median (range [IQR]) or absolute numbers (%).

### Patient selection

Patients undergoing elective, routine anesthesia were asked to participate in this trial. Exclusion criteria were: age under 18 years, potential risk of regurgitation, pregnancy, ASA classification > III and patients with an anticipated difficult airway.

### Setting and intervention

All intubations were performed using either a size 3 curved blade (“control”) or a size 3 curved blade with a tube-guiding channel (“channeled”), respectively. The size of the ETT was predefined by the standard operating procedure of the study center. ETT size 7.0 internal diameter (ID) was used for female patients and 7.5 ID for male patients (Mallinckrodt Medical, Athlone, Ireland). For intubation in the control group a malleable stylet was inserted in the ETT in a hockey-stick shape (distal end of ETT angulated of 90°). When applying the channeled blade, the ETT was preloaded in the tube-guiding channel before insertion of the channeled into the mouth of the patient. After three minutes of pre-oxygenation with a facemask, anesthesia was induced with sufentanil (0.2–0.5 μg.kg^-1^) and propofol (2–3 mg.kg^-1^). Mivacurium (0.2 mg.kg^-1^) or atracurium (0.5 mg.kg^-1^) was used for neuromuscular blockade. The intubation attempt was performed three minutes after injection of the neuromuscular blocking agent.

### Outcome measurements

Our primary study objective was to determine whether there is a difference in intubation time (seconds) for the control compared to the channeled blade. The intubation time defined when the blade tip passed the incisors to the point until confirmation of the first wave of CO_2_ of the capnometer (Primus, Dräger, Lübeck, Germany). Additionally, two time points before final tracheal placement were evaluated: time to “glottic view” and time to “tube placement” after introducing the device into the oral cavity. “Glottic view” was defined from blade insertion until the best view on the glottis was achieved. “Tube placement” was defined as time from inserting the ETT until the tip of the tube disappeared between the vocal cords. Time was measured using the built-in stopwatch of the monitoring system (Philips^®^ MX500, Philips GmbH, Hamburg, Germany).

Secondary outcomes were: first attempt and overall intubation success rates, visualization of the glottis using Cormack & Lehane grade (C&L; [13]) and percentage of glottic opening score in percent (POGO; [14]). External laryngeal manipulations like the BURP manoeuvre (backwards, upwards and rightwards pressure) were allowed to improve glottis view. Failed intubation was defined as: a) an elapsed intubation time of more than 120 s; b) failed tracheal placement of the tube; and c) removal of the device / repositioning from the oral cavity without advancing the tube. After each intubation, the anesthetist was asked to rate the degree of difficulty of intubation using a 5-point Likert scale (1 = very easy until 5 = very difficult; [15]) for each blade. Patient data (age, gender and BMI) were also documented.

### Sample size and statistics

Sample size calculation was performed based on results from a previously published manikin study [16]. In this publication, participants needed an average time of 60 s (median) (range [11–60]) to ventilate using the control, compared to 20.5 s with the channeled blade (range [7.2–60]). Using these data for sample size calculation, a total of 46 patients were considered necessary to prove a local 5% level with power >90%. Data were tested using a Log-rank test. All recorded data were documented using a controlled evaluation sheet. Patients were randomized to treatment group using GraphPad QuickCalcs Web site: http://www.graphpad.com/quickcalcs/randmenu (accessed January 2015).

For statistical analysis, GraphPad Prism (Vers. 6.0 for MAC; GraphPad Software, San Diego, CA, USA) was used. Data were expressed as the median (range; interquartile range [IQR]) for non-Gaussian variables. A chi-squared test was used to compare the success rate. Comparisons the view of the glottis and the Likert-scale were analyzed by the Mann-Whitney U test. The differences were considered statistically significant if the p-value was less than 0.05.

## Results

From February to March 2015, a total of 46 (control n = 23; channeled n = 23) adult patients eligible to participate in this study were included (Fig 1 and S3 File). No statistically relevant differences in patient demographics were found (Table 1; *p*>0.05). More residents with fewer than four years of clinical experience than anesthesia specialists participated in this study (*p*<0.001). The provider experience in anesthesia practice was comparable between the two groups. (*p* = 0.82).

### Success rate and intubation time

All intubation attempts were successful with a maximum of two attempts and were completed within the time limit of 120 s. Correct endotracheal placement of the control blade was successful at the first attempt in 23/23 (100%) patients, compared to 22/23 patients with the channeled blade (96%; *p* = 0.31).

The overall time needed for first ventilation was shorter with the control 40 s [24–58], compared to the channeled 59 s [40–74] (*p* = 0.03).

**Fig 3:**
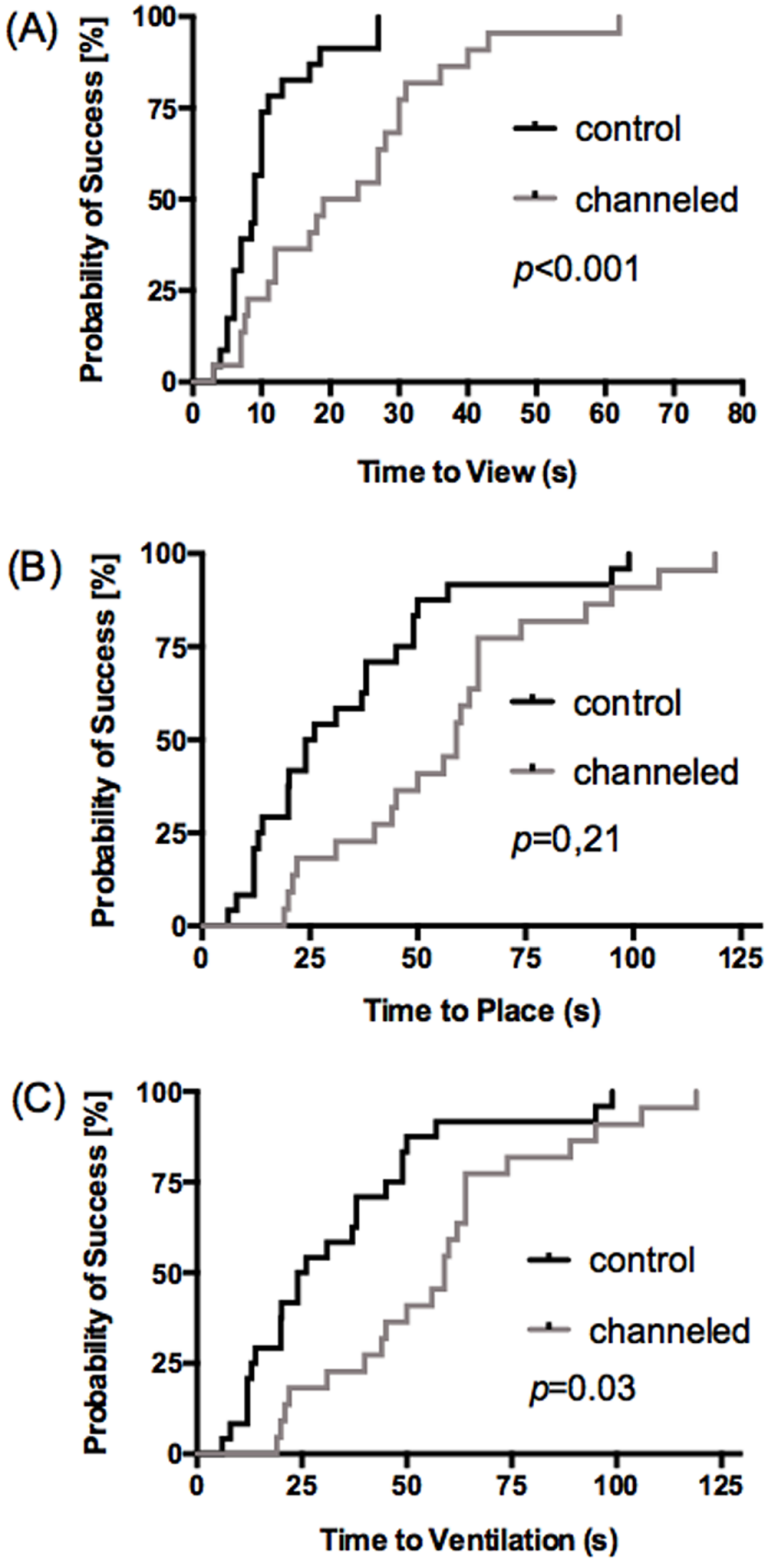
Time sequnce of intubation. **Time steps** of (A) differences in Time to view (B) differences in Time for place the TT (C) differences in overall intubation time; Data are given in Kaplan-Meier curve and median.

Time to view with 9 s [6–11] was significantly shorter with control (*p*<0.001). However, there were no differences between time to place with control 26 s [13–49], compared with channeled 44 s [21–54] (*p* = 0.21). Fig 3 shows the chronological sequence of the time points evaluated.

### Visualization of the glottis

Glottic view was excellent in both groups: control C&L I [I], POGO 100% [100] and channeled C&L I [I] (*p* = 0.48), POGO 100% [100] (*p* = 0.55).

### Subjective assessment of blades

Using the 5-point Likert-Scale, no differences between groups were observed. The degree of difficult intubation was evaluated with the control as 2 (easy) and with the channeled as 3 (moderate) (*p* = 0.05).

## Discussion

In the present study, a faster intubation time using a curved video laryngoscopic blade was found in a group of 46 patients. Using a similar shaped laryngoscope blade with a tube-guiding channel did not have any advantages compared to a similar blade without the channel. In this trial, intubation success rate, visualization of the glottis and subjective assessment were in this trial comparable between groups. So far, most studies have compared the King Vision with other makes of VL or the traditional Macintosh laryngoscope [16–22].

Patient demographics and provider data were comparable to other studies investigating the King Vision [16,19–21,23]. The success rate in this trial was higher than in two other studies comparing control with channeled blades or with other video laryngoscopes, but still lay within the range of the published data [14,18–20,23]. In this one failed case the performer had an anesthesia experience of 13 months and 15 video laryngoscopic intubations prior to the study. The examination of the patient showed no predictors for a difficult airway (Mallampati class I, thyromental distance > 6.5 cm). The laryngeal view was rated C&L grade II and with a POGO of 20%. Using the channeled-blade the ETT moved towards the right part of the larynx and could not be steered towards the vocal cords. After the intubation time limit of 120 seconds was reached the intubation attempt was stopped.

Overall, intubation success rate for the KV VL, varies in several studies and ranges from 51% to 100% [17, 18, 19, 20]. In one study comparing channeled King Vision with non-channeled and Macintosh blade, the overall success rate of intubation was better with the channeled (87%), compared to the non-channeled blade (47%) [16]. In several studies, most of the participants were paramedics or registered nurses with practical video laryngoscopy experience of less than 10 uses [17,19,22,23]. The study setting in these publications was also different in subjects (manikin or human cadaver vs. alive patients) and in the study setting (pre-hospital setting with anticipated difficult airway) [17,22,23]. We speculate that previous experience with laryngoscopy and especially other types of non-channeled video laryngoscopes results in a better success rate. Additionally, stylet use has been reported to increase the success rate with video laryngoscopy.

**Fig 4:**
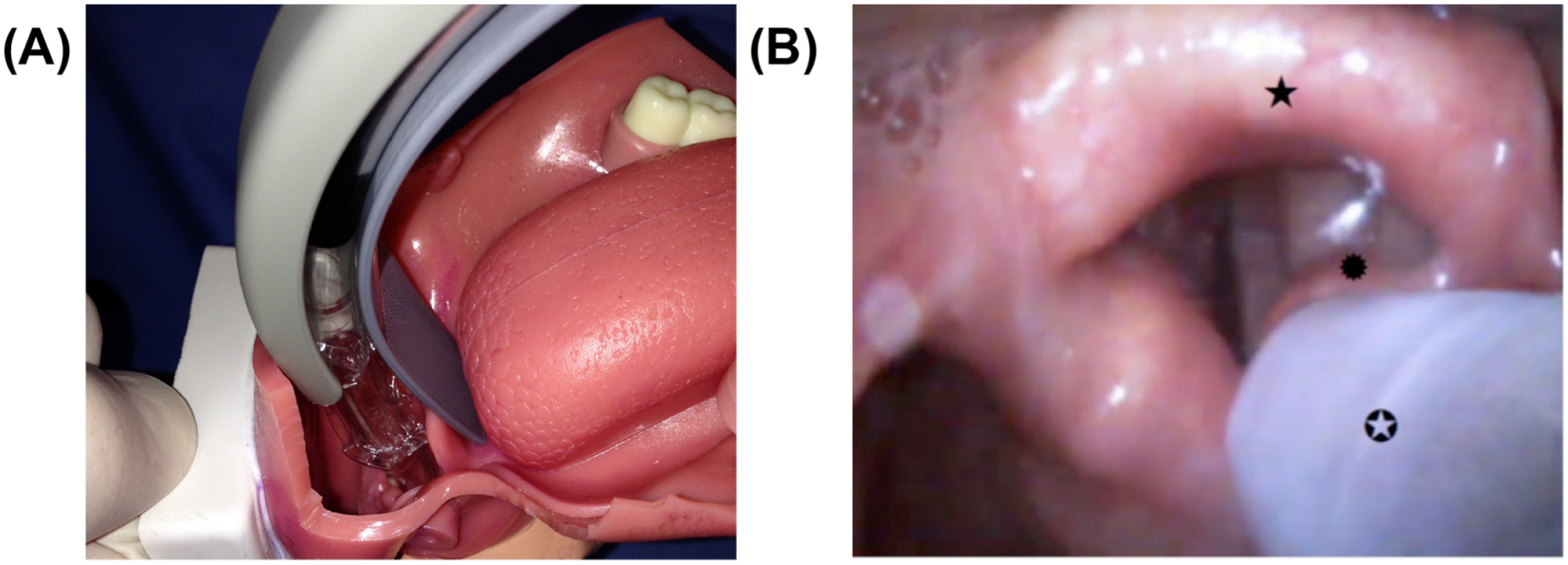
Insertion technique and view. (A) typical Macintosh-blade technique: blade tip placed in the vallecula (B) view from the monitor display using the channeled blade (★: epiglottic; ✪: ET leave the channeled; ✹: right arytenoid).

The primary endpoint of this study was evaluation of the time for first ventilation confirmed by the first CO_2_-wave on the capnometer. Most studies cannot be compared because the definition of “intubation time” varies. In two studies, the intubation time with the channeled blade was 20.1 s [20] compared to 25 s [17] evaluated in a manikin. The time required in adult patients was in median 38 s when using a channeled blade [20]. In one study comparing both KV blades in a manikin, the time for first ventilation with the non-channeled blade was 60 s [11]. The measurements differ between these two studies from taking hold of the handle of the device until confirmation of adequate lung ventilation [16] and the time stops when the cuff of the ETT is inflated [23]. Both studies used a manikin (Laerdal, Skill Trainer), which is suitable to evaluate and practice with a novel device since the airway is controlled (i.e. mouth already open, spacious mouth cavity, epiglottis fixed in the pharynx). It is likely that the intubation time in human patients undergoing general anesthesia in which anatomy varies is prolonged. Time for first ventilation was prolonged using the channeled blade in this study, presumably because advancing the tube meant that the tip did not follow the line of the blade (Fig 4B). The tube contains the channel and drifted towards the right arytenoid. Because of this deviation, it was necessary to correct the position of the blade multiple times until it was possible to advance the tube through the glottis. Based on the manufacturer`s instructions, the blades allows either a Macintosh blade-like technique (Fig 4A) to position the tip of blade in the vallecula or a Miller blade-like technique (the blade tip passes underneath the laryngeal surface of the epiglottis) to be used successfully. In order to achieve optimal intubation technique, it is recommended to position the KV blades with the tip in the vallecula (Macintosh blade-like technique). In this trial, a Macintosh-approach was also used. However, we observed that inexperienced participants initially positioned the tip of the channeled blade too deep in the airway, which resulted in uploading of the epiglottis (Miller blade-like position). In this position, steering of the tube towards the glottis was nearly impossible because the channel of the blade restricted tube movement.

The Miller blade-like technique, with an upload of the epiglottis, results in difficult handling when directing the ETT opposite the glottis. This might influence the longer time taken for intubation with the control or channeled blade compared with other published results.

It is generally thought that the learning curve for video laryngoscopy is steep. A minimum of 10 uses is necessary to improve the success rate or intubation time with the C-MAC^™^ Macintosh-blade (Karl Storz, Tuttlingen, Germany), McGrath Series 5 (Aircraft Medical, Edinburgh, UK) and GlideScope^™^ Ranger (Verathon, Bothell, USA) [23–25]. Comparative studies analysed other tube-guiding blades like the Airtraq^™^ (Prodol Meditec S.A., Vizcaya, Spain) or the Pentax^™^ AWS^®^ (Hoya Corporation, Tokyo, Japan), presenting different results between 5–50 applications until a success rate >80% is achieved [8,26,27]. This skill level of the operator determines the efficacy of success intubation. Also, training and experience using manikins alone does not enable the optimized use of any device, even when experienced airway specialists considered the training sufficient. Significant clinical experience was proposed before using Airtraq channeled blades in an emergency intubation setting [23]. In a recent study, a significantly higher number of 76 uses were documented for the GlideScope VL to master its use, indicating that the learning curve might be flatter than originally thought [28]. The first-pass success rate using highly curved blades ranges between 74 and 96% [25,27,29,30]. There are two typical difficulties of the ETT placement. One is that the tongue interferes with the advancing of the ETT, while the second is that the high curvature of the blade typically makes it virtually impossible to obtain a direct view of the glottis. To overcome the high curvature of the blade with an ETT, a malleable stylet might be necessary to shape the ETT according to the blade shape for VL with hyper-angulated blades. However, using a stylet bears the risk of causing airway injury, especially to the soft tissue of the pharynx [31,32]. A current method is to shape the ETT at 60 to 90 degrees at the distal curvature and rotate the ETT after passing the vocal cords by about 180 degrees down and forward [33,34]. This feature could prolong the intubation time and reduce the intubation success. Because of potential risk of upper airway injury, it is important to advance the ETT beyond the uvula under direct vision before directing one’s attention to the video monitor [12].

When comparing the visualization of the glottis, we found no differences between the control and the channeled blade using the Cormack & Lehane scoring system and POGO-score. These findings are similar to one manikin study, which evaluated a C&L grade I (86%), II (14%) and best view of the glottis entrance from 100% by a rate of 27 [23]. This was an expected result in this trial since both blades examined are curved blades, with the only difference being that the tube is pre-loaded in the channeled blade.

Based on subjective judgement, after completing the study, the participants rated the use of the control as very easy to easy and the channeled blade as easy to moderate. Our findings are contrary to another manikin study, in which the control was rated from difficult to very difficult [11]. In contrast to this study, participants were registered nurses without a high level of expertise in tracheal intubation. A reason for preferring a channeled blade might be that preloading of the ETT makes it easier for more unexperienced providers to guide both the tube and the blade tip towards the glottis. The intraoral space created by laryngoscopy with a curved blade might be limited and additional manipulation with the ETT loaded on a stylet might be more difficult. Thus, this might contribute to a higher Likert Scale score. The reason for this subjective rating in this study might be the high pre-existing experience with video laryngoscopy using curved blades without a guidance channel.

There are several limitations in our study. First, advancing the tip of the channeled blade too far towards the glottis might not leave enough space to allow directing the tip of the ETT through the glottis. Second, this trial might be limited by the heterogeneous experiences of the providers (e.g. experiences in anesthesia practice and in video laryngoscopy technique). Some providers had a high level of expertise, which might have influenced the results of this trial. Most of the participants had a higher experience using the video laryngoscopy with a non-channeled blade and a stylet with angulated hockey stick tip to insert the ETT into the glottis. Third, the standardized tube size of this trial could result in a limited generalizability for the intubation time when a larger tube size is used. Until recently, the use of a nerve stimulator to confirm sufficient neuromuscular blockade was not standardized in the study center. Because of the design of this trial, the protocol did not allow blinding of the operators using the different blades. Finally, only one VL with a hyper-angulated blade was used. The KV is the only device that uses the same type of angulated blade for channeled and non-channeled. Consequently, this device was evaluated to compare channeled vs. non-channeled blades.

## Conclusion

Practitioners with experience in clinical practice and the technique of video laryngoscopy facilitated tracheal intubation without any esophageal intubation. We found for the primary endpoint, that a curved video laryngoscopic blade was superior in intubation time compared to a curved blade using a tube-guiding channel. The use of a VL with tube-guiding channels does not optimize the performance of the tracheal intubation of anesthesiologists in a controlled environment.

## Supporting information

S1 File CONSORT statement. (DOC)

S2 File Study protocol King Vision. (DOCX)

S3 File Data availability. (XLS)

## Abbreviations

ASA: American Society of Anesthesiologists
BMI: Body Mass Index
BURP: Backward upward rightward pressure
channeled: King Vision channeled blade
control: King Vision non-channeled blade
EMS: Emergency Medical Service
ENT: Department of Ear, Nose, Throat, Neck & Head surgery
ETT: Endotracheal tube
VL: Video laryngoscope
