# Supplementary material for: Using King Vision video laryngoscope with a channeled blade prolongs time for tracheal intubation in different training levels, compared to non-channeled blade
Source: PLoS One. 2017 Aug 31;12(8):e0183382. doi: 10.1371/journal.pone.0183382 (PMC5578637; doi:10.1371/journal.pone.0183382)
Supplement: S2 File — (DOCX) [file pone.0183382.s002.docx]

**Study Protocol**

**Using King Vision video laryngoscope with a channeled blade prolongs time for tracheal intubation**

**King Vision-Study**

Principal Investigator:

Name Marc Kriege, MD

Adress Langenbeckstrasse 1, 55131 Mainz

Phone +49 (0) 6131 17- 0

Fax +49 (0) 6131 17- 6209

E-Mail makriege@uni-mainz.de

Study Nurse

Name Susanne Mauff

Adress Langenbeckstrasse 1, 55131 Mainz

Phone +49 (0) 6131 17-7175

Fax +49 (0) 6131 17-6209

E-Mail susanne.mauff@unimedizin-mainz.de

Statistic

Name Irene Schmidtmann, DSc

Adress Obere Zahlbacher Str. 69, 55131 Mainz

Phone +49 (0) 6131 17-3951

Fax +49 (0) 6131 17-2968

E-Mail irene.schmidtmann@uni-mainz.de

This study protocol is a confidential communication and is solely the guidance for the clinical study. It should individuals and institutions that are not involved in the clinical trial, not be made available and can be used without written consent of the principal investigator for any other purpose.

**Abbreviations**

| **CRF** | Case Report form |  |
| --- | --- | --- |
| **GCP** | Good clinical practice |  |
| **ICH** | International conference on harmonization of technical requirements for registration of pharmaceuticals for human use |  |
| **ISF** | Investigator site file |  |
| **ITT** | Intention to treat |  |
| **SAP** | Statistical Analyze Plan |  |
| **TMF** | Trial master file |  |
| **ETT** | Endotracheal tube |  |
| **VL** | Videolaryngoscopy |  |
| **C & L** | Cormack and Lehane | |
| **POGO** | Percentage of glottic opening | |
| **VAS** | Visual Analog scale | |
| **CO_2_** | Carbon Dioxide | |
| **OSAS** | Obstructive Sleep apnea Syndrome | |
| **SOP** | Standard Operating Procedure | |
| **CCD** | Charged Coupled Device | |
| **CMOS** | Complementary Metal Oxide Semi-conductor | |
| **RCA** | Radio Corporation of America | |
| **LED** | light emitting diode | |

**Study Protocol Overview**

| **Title** | Using King Vision video laryngoscope with a channeled blade prolongs time for tracheal intubation |
| --- | --- |
| **Short Title** | King Vision-Study |
| **Principal Investigator** | Marc Kriege, MD |
| **Study Protocol-Code** | 1.1 |
| **Indication** | Patients having elective surgery with general anaesthesia and requiring mechanical ventilation via an endotracheal tube are recruited |
| **Primary Endpoint** | Comparison of the overall intubation time between the Channeled and the Standard Blade |
| **Secondary Endpoints** | Time Sequences:  1. Time to View of the Glottic  2. Time to Place the ETT  3. Time to Ventilation  4. View (C&L, POGO)  5. Five Point Likert Scale: 1 very easy –  5 very difficult |
| **Study design** | Prospective, controlled, monocenter, randomized |
| **Patient Selection** | Inclusion criteria:   - Age ≥ 18 Years - Elective surgery   Exclusion criteria:   - Pregnancy - ASA Class IV - Participation in other Studies - Unable to provide informed written consent - Risk for aspiration - Above one predictor for an anticipated difficult airway |
| **Duration of study** | The first patient expected in May, 2015. Completion of the last patient at November 2015. Duration of the study: 6 months. |
| **Number of patients** | 44 (22 Patients for each group) |
| **Statistics** | The respective times until first ventilation analyzed with the Wilcoxon test. Time to view/ -placed the ETT tested using the t-test. For all statistical tests, p values of 0.05 or less will be taken to indicate a significant difference. |

| **Accountability**   \| **Principal Investigator** \| Name Marc Kriege, MD  Adress Langenbeckstrasse 1, 55131 Mainz  Phone + 49 (0) 6131 17-0  Fax + 49 (0) 6131 17-6209  E-Mail [makriege@uni-mainz.de](mailto:makriege@uni-mainz.de) \| \| --- \| --- \| \| **Study Nurse** \| Name Susanne Mauff  Adress Langenbeckstrasse 1, 55131 Mainz  Phone + 49 (0) 6131 17-7175  Fax + 49 (0) 6131 17-6209  E-Mail [susanne.mauff@unimedizin-mainz.de](mailto:susanne.mauff@unimedizin-mainz.de) \| \| **Statistic** \| Name Irene Schmidtmann, DSc  Adress Obere Zahlbacher Str. 69, 55131 Mainz  Phone +49 (0) 6131 17-3951  Fax + 49 (0) 6131 17-2968  E-Mail [irene.schmidtmann@uni-mainz.de](mailto:irene.schmidtmann@uni-mainz.de) \| |
| --- | --- | --- | --- | --- | --- | --- |

**Index**

| 1 | Introduction | 9 |
| --- | --- | --- |
| 1.1 | Background | 9 |
| 1.2 | Rationale for the clinical study | 9 |
| 1.3 | Risk-benefit ratio | 10 |
| 2 | Objectives | 11 |
| 2.1 | Primary Endpoint | 11 |
| 2.2 | Secondary Endpoint | 11 |
| 3 | Study Design | 12 |
| 3.1 | Timeline | 12 |
| 3.2 | Number of Patients | 12 |
| 3.3 | Eligibility | 12 |
| 3.4 | Early termination of the study | 13 |
| 4 | Intervention | 14 |
| 5 | Statistic | 15 |
| 5.1 | Sample Size | 15 |
| 5.2 | Analysis of the primary outcome | 15 |
| 5.3 | Analysis of the secondary outcomes | 15 |
| 5.4 | Patient analysis | 15 |
| 6 | Quality assurance | 16 |
| 6.1 | Document management | 16 |
| 6.2 | Archiving | 16 |
| 6.3 | Audits | 16 |
| 7 | Data administration | 17 |
| 7.1 | Responsibility | 17 |
| 7.2 | Data collection | 17 |
| 7.2.1 | Paper-based Data collection | 17 |
| 7.2.2 | Electronic Data management | 18 |
| 7.3 | Patient identification list | 18 |
| 7.4 | Data storage | 19 |
| 8 | Administration | 20 |
| 8.1 | Study Intervention | 20 |
| 8.2 | Changes in Study plan | 20 |
| 9 | Ethical aspects | 21 |
| 9.1 | Good clinical practice | 21 |
| 9.2 | Patient consent | 21 |
| 9.3 | Confidentiality | 22 |
| 9.4 | Professional order for physicians | 23 |
| 9.5 | Responsibility for the Principal Investigator | 23 |
| 9.6 | Publication | 23 |
| 10 | Signatures | 24 |
| 11 | Declaration of the Principal Investigator | 25 |
| 12 | References | 26 |

**1. Introduction**

**1.1 Background**

The King Vision® VL (King Systems Corporation, Noblesville, IN 46060, USA; German sales by Ambu® GmbH, Bad Nauheim, Germany) is used in preclinical and in-hospital airway management since its market introduction 2013. The tracheal intubation using a direct laryngoscope requires a alignement of the oropharyngeal axis and the technique is difficult to learn (1,2). The King Vision® Vl consists of a 2.4-inch reusable Display on the handle with battery compartment for three AAA batteries (1.5 V) to the power line and the end of the stylet with a OLED (Organic Light Emitting Diode). At the lower end of the stylet is a CMOS chip and an LED light source. By CMOS chip technology, an automatic white balance and automatic focusing is performed (3). Using anti-fogging solution before use of the instrument omitted. The image transfer can be projected through an RCA connector (standard video input) to an external monitor. The channeled blade should facilitate to place the ETT, therefor no stylet is required. With the non-channeled blade, the intubation is facilitated by using a flexible stylet (4). In one manikin study, the King Vision® channeled blade compared with the standard blade (4). The learning curve using the King Vision VL is rapid, six applications using the standard blade and 4 applications with the channeled blade (4).

**1.2 Rationale for the clinical study**

So far the King Vision® standard blade compared with the channeled blade is only evaluated in manikin studies. Clinical trials with anesthized patients are missing in the database. The channeled blade brings likely through its preloading of the ETT in the guided channel and the positioning in front of the glottis, a possible time advantage. When using the standard blade, the ETT with a stylet can only placed after visualization of the glottis. This procedure is standardized in handling with the video laryngoscopy without tube guiding channels.

The primary endpoint in this proposed study is the time to ventilation, as measured by the apparent end-tidal CO_2_ wave on the anesthesia ventilator, of the standard and the channeled blade.

The Establishment of VL with different Blade systems and camera chip technology has improved in recent years, thanks to technical improvements and rigorous clinical observation, the airway management in anesthesia and intensive care. Thanks to this innovative technology, situations in which no positioning of the endotracheal tube are possible, become less frequent. The comparative analysis of these two studies blades are used for an optimization of the patient safety (shorter intubation time, minimizing damage to teeth / soft tissue). Secondly, the test results generated under standardized conditions should provide an impetus to other hospitals with regard to economic aspects that may contribute to the purchase of the two devices. Currently, only 4 - 7% of all German hospitals using Videolaryngoscope in their daily clinical routine (Stand at moment).

**1.3 Risk- benefit ratio**

Both devices are used according to their authorization of the manufacturer and study personnel are familiar with their use in the daily routine. The anesthesia implementing performed according to the SOP of the Department of Anesthesiology and there are no specific risks above the normal anesthesia routine. Furthermore, the time for anesthesia not prolonged through the study-related activities. There are no changes in this clinical trial neither the treatment or medication of the patients.

The incidence of teeth injury is frequently lower in contrast to the conventional laryngoscopy (5,6).

The principal investigator will be informed about all the details of relevant and new results, including adverse events related to study treatment.

**2. Objectives**

The aim of this study is to compare the King Vision® standard blade with the channeled blade with respect to their time to first ventilation, view of the glottic and evaluation about the handling.

**2.1 Primary Endpoint**

The primary endpoint of the study involves the comparison of the two blades in terms of time to the first ventilation (shown after three breaths through an end-tidal CO_2_ - wave on the anesthesia ventilator). The time limit for the intubation attempt amounts to 120 seconds.

**2.2 Secondary Endpoints**

Comparing the respective times of both devices for:

1. Time to View of the glottis (defined by removing the mask for

preoxygenation to the individual view of the glottis)

2. Time to Place of the ETT into the trachea (defined as the time from the point

of view of the glottis until disappearance of the black marker of the ETT

behind the glottis)

3. Time to Ventilation (shown after three breaths through an end-tidal CO_2_ -

wave on the anesthesia ventilator

3. Visualization of the laryngeal structures (C & L grade and POGO scale score)

4. Assessment of both blades by the user (using the 5 point Likert Scale: 1 very

easy - 5 very difficult)

**3. Study Design**

This is a prospective, single-center, randomized, controlled study. The randomization of study participants carried out centrally by the Study site of Department of Anesthesiology, University Medical Mainz, Germany by a 1:1 Randomization (QuickCalcs GraphPad, La Jolla, CA, USA).

**3.1 Timeline**

The Clinical Trial will begin after receipt the positive votum of the ethic committee. At this moment the clinical trial will likely during 6 Months (May - November 2015). Both the current total length of the study as well the period of recruitment of the patients can vary.

**3.2 Number of Patients**

44 participants (22 patients for each group) are planned to include in the clinical trial. Each Patient will be included only one in the study.

**3.3 Eligibility criteria**

**Inclusion criteria**

Patients in which all of the following criteria are met, can be included in the clinical study:

- Age ≥ 18 Years
- elective surgery

**Exlcusion criteria**

- Pregnancy
- ASA Class IV
- Participation in other Studies
- Unable to provide informed written consent
- Risk for aspiration
- Above one predictor for an anticipated difficult airway

**3.5 Early termination of the study**

For the following reasons , the entire clinical trial might be canceled at the discretion of the principal investigator :

- Occurrence of unknown, adverse events or events with considerable frequency and severity
- Medical or ethical reasons, the adverse effect on the further implementation of the clinical trial
- Inadequate recruitment of study participants
- For each study participant / patient participation in the study will be canceled under the following circumstances :
- Unexpected emergency or situation under a Difficult Airway

Management

- Verbal withdrawal of consent for study participation by the patient
- Patients who discontinue study participation prematurely replaced

The ethics committee must be informed.

**4. Interventions**

**Concomitant treatments in both groups**

Before the start of patient recruitment, the study personal accesses the eligibility criterias and the patients are evaluated for factors known to predict diffult intubation (e.g BMI, mouth opening, thyromental distance, Mallampati score). The results of this evaluation are recorded in a CRF sheet. Afterwards its necessary to get a written informed consent of the patient.

**Day of Surgery**

Preoxygenation is achieved using the Standard Operating Procedere of the University Medical Center Mainz. Anesthesia is then induced by injecting anesthetic agents (Opioid, hypnotic-/ and neuromuscular blocking agent). The type and dosage of these drugs are to consider of the intubating physician. Laryngoscopy is performed using the blade allocated at random:

- King Vision standard blade
- King Vision channeled blade

The size of the ETT (7.5 Internal Diameter) is always similar in both groups. The time to ventilation starting by each introduction of the King Vision VL into the oral cavity. The endotracheal tube position is confirmed by analyzing the capnography curve. The overall time to ventilation is defined as the time from inserting the blade into the oral cavity to observation of the first inflection on the expired capnography curve on the anesthesia respirator. The C & L grade of glottis visibility and the POGO scale score are evaluated simultaneously on the monitor display of the King Vision VL. After Intervention the physician asked for his subjective Assessment of the handling with the blade (by means of the Likert scale)

**5. Statistic**

**5.1 Sample Size**

On the basis of previous data (4,7) we expected a difference of five seconds in time to ventilation in the channeled blade group. Assuming that using channeled blade abbreviated the time to ventilation with a Power of 90 %, 22 patients are needed in each group, that is, 44 patients at all.

**5.2 Analysis of the primary outcome**

All data were exported from our CRF into MS Excel (Microsoft, Redmond, WA, USA) for analysis. Statistical testing was performed using Prism 7 (Graph Pad Software, La Jolla, CA, USA). The time to ventilation will be compared between the two groups using the Wicoxon-Mann-Whitney-test. A p-value of <0.05 was considered significant.

**5.3 Analysis of the secondary outcomes**

Secondary outcomes will be using a two-tailed Fischer ’s exact test for categorical data and an unpaired t-test to compare continuous data.

**5.4 Patient analysis**

Only patients in whom the intervention was fully carried out, evaluated in the Statitistical testing. (Per protocol Analysis)

**6. Quality assurance**

**6.1 Document management**

The data quality assurance and monitoring of studies - enforcement at the center must be guaranteed by statutory regulations. Mandatory for investigators it is necessary authorized third party, such as Monitors to grant auditors and inspectors to inspect the original medical records of participating in the study patients. These patient-related source data include all paper-based and electronic documents, such as Entries in SAP®, COPRA® or the laboratory program LAURIS®. The insight acquiring persons subject to the obligation of secrecy. The patient approved his grant for consent to participation in the study this inspection in its original medical records through above authorized personnel .

**6.2 Archiving**

After completion of the clinical study, all study materials are kept in the Study Center of the Department of Anesthesiology. It is the responsibility of the principal investigator to ensure that you retain the patient identification lists at least 15 years after completion of the clinical trial. All original medical records of study participants must be retained either as required by the respective rules of the University Medical Center Mainz.

**6.3 Audits**

Competent authorities may require access to all source documents, case report forms and other documentation study for a center audit or inspection. The investigator must guarantee direct access to these documents and support these activities at any time. Medical records and other study documents can be copied during an audit or inspection, provided that the patient name be omitted from the copies to ensure confidentiality .

For this clinical study, no audits are planned .

**7. Data administration**

**7.1 Responsibility**

The Principal Investigator is responsible for the Data administration in this purpose clinical trial:

Marc Kriege, MD

Langenbeckstraße 1

55131 Mainz, Germany

**7.2** **Data collection**

**7.2.1 paper-based Data collection**

For each patient a data collection sheet (CRF) was developed. This is one place with all the other findings of the patient file to source data, because it contains information that is collected directly on the patient and not be verified on the basis of the patient record. The CRF includes the following information:

• Screening of patients preoperatively

• Data collection intraoperatively

All study data of a subject to be documented in his study and acts in his CRF. The full and correct completion of all parts of CRFs is up to the investigator or to its authorized representative. Likewise, he must ensure that the entries of the CRFs can be checked against the source data. Any error should be crossed out with a simple line such that the original record remains legible. The correction of the entry should be in addition to this added together with the signature of the study physician, the date and the reason for the correction. If the principal investigator other persons authorized completed CRFs, he must announce the names, positions, signatures and initials of people in the study line. The study doctor or a person authorized by him should fill the pages of CRFs to visit, as soon as possible to collect. Preferably, the completing the CRFs happens on the day on which the subject was seen to an investigation or other study-related appointment. Any missing items must be completed for final examination at the latest. For missing data an explanation should be given. The entries in the CRF-sheets must be authorized by the responsible investigator to study end by date and signature. The original of the CRFs remains in the trial record of the patient and is thus kept in the study center.

**7.2.2 electronic Data collection**

In addition to the paper-based form, this study is documented electronically. For this purpose any information from the trial record and the CRF are either scanned or transferred to the computer in a tabular form .

The database management system is capable of accurate and complete copies of the data to create in visual form for inspection by authorities or ethics committees .

**7.3 Patientenidentifikationsliste**

Each patient is assigned to an identification number. These create a document containing the full name , date of birth and sex of the patient and it identifies with an identification number (patient identification list). This pseudo-nymization done for the process of data transmission in electronic form. In order to identify patients for future demands, this list is maintained and kept for at least 15 years after the trial. The analysis and publication of data is then only in anonymous form .

**7.4 Data storage**

All information in electronic format are backed up regularly. It is ensured that the anonymity of the patient is ensured in the analysis. All study data including the data recorded electronically archived and kept for at least 15 years after completion of the study. This concerns:

- Original CRFs
- Study Protocol
- Patient identification list
- Signed informed consent
- Study Files
- Original questionnaires
- Final report

Should other laws require a longer retention period, this must be considered in any case .

**8. Administration**

**8.1 Study interventions**

The planning, execution and evaluation of the study is a subject to the require-ments of ICH-GCP and the applicable regulatory requirement.

As protocol violations to understand all deviations from the instructions and procedures described in this study protocol. Examples for this are:

• Lack of data collection or carrying the wrong time

• Lack of Compliance

• violation of inclusion or exclusion criteria

It is the responsibility of the investigator to avoid protocol violations in order to keep the patient after he was included in the study.

It is the duty of all study participants to communicate all protocol violations immediately to the principal investigator and documented. Before statistical analysis which are then discussed with the responsible biostatistician.

It must also be secured by the investigator that all collected from the patient data is documented in the test plan. Minor discrepancies are certainly not to be avoided in everyday work, however, must be documented with justification.

**8.2 Changes in study plan**

Subsequent changes to the agreement valued by the ethics committee clinical trial may be made only if the change was evaluated by the ethics committee approval. This includes the following changes :

- Changes that affect the safety of the patient
- Additional data collection or analyzes that require a change in the patient information and / or patient consent
- Interpretation of the scientific documents in the clinical trial is based or the scientific validity of the study results will be affected
- Significant changes in the nature of management or conduct of the study

Written by fixing Note to File by the principal investigator is sufficient for smaller not requiring approval study plan amendments and are stored in the TMF. Without authorization are changes that do not fall under the above points.

**9. Ethical aspects**

**9.1 Good clinical practice**

The requirements of the ICH Guideline for Good Clinical Practice (GCP) E6 of June 1996 and CPMP / ICH / 135 /95 of September 1997 is in addition to the national laws and the Declaration of Helsinki (Version Sommerset West 1996) the basis for the implementation of this study ,

All study contributors (principal investigator, Agents, auditors etc. ) are obliged to participate in this study, according to these specifications .

**9.2 Patient consent**

Before being incorporated into the clinical trial, patients must consent to participate after kind, scope and possible consequences of the clinical trial were them, explained to them in an understandable way.

The document must be written in a language understandable for the patient and must state the name of the person who conducted the informed consent discussion. Under German law, the person who educates the patient must be a physician.

The patient must be given a copy of the signed Patient Information and -informed consent. The investigator shall retain the original signed the consent form.

The study personnel may not carry out trial-specific action before a valid consent has been obtained.

After reading the patient information and informed consent of the patient must declare his consent in writing. The consent of the patient must be confirmed at the time of consent by personally dated signature of the patient and personally dated signature of the person who conducted the informed consent discussion.

**9.3 Confidentiality**

The requirements of the Federal Data Protection Act (FDPA), taking into account the relevant data protection legislation into consideration.

Patient names and other confidential information are subject to medical confidentiality and the provisions of local data protection regulations. During the study, patients will be identified only by individually identifiable codes (e.g patient number). The data stored on a computer study surveys are stored in accordance with local data protection regulations. To protect this data organizational procedures are implemented to prevent the transfer of such information to unauthorized persons. The relevant regulations regarding the privacy laws are therefore fully met.

Patients are informed that representatives of the principal investigator or authorities shall take any medical records to verify the data collected. All personal information will be disclosed for inspection are handled most strictly confidential and as specified by the local data protection regulations.

The principal investigator performs an identification list of his patients (identification numbers and the patient's name) so that the records can be identified.

Should a patient withdraw his consent, checks the student administration whether it has stored data are still needed. If this is not the case, these data will be deleted immediately. All personal data will be deleted after 15 years of archiving, unless other contractual or legal regulations require longer retention periods.

**9.4 Professional order for physicians**

According to the specifications of the professional order for physicians §15 (German law) the study will be submitted for review prior to the relevant ethics committee. The study will be initiated only if the favorable opinion of the ethics committee is present .

Subsequent changes to the study protocol for ongoing studies are done via amendments. The principal investigator is responsible for obtaining the favorable opinion of the ethics committee for the amendment .

The study commission shall keep all correspondence with the Ethics Committee and the competent authorities. The ethics committee must be regularly submitted reports on the course of the study, if not specified otherwise in the ethics committee .

The Ethics Committee is informed of the end of the study .

**9.5 Responsibility for the Principal Investigator**

The Principal Investigator ensures that all individuals involved in the study are sufficiently informed about the study protocol, protocol amendments, study treatments, study-related duties and functions. The responsible study physician maintains a list of study doctors and other appropriately qualified personnel, to which it has delegated significant study-related tasks. Any kind of changes through authorized study personnel will be reported without delay to the Ethics Committee of the Medical Association Rhineland-Palatinate.

**9.6 Publicationen**

Any publication of the results of this clinical study, which is presented by the study personnel, his authorized representative or by the principal investigator, either partially or entirely as an article in (professional) journals , must approved by Marc Kriege. It is planned to publish the results of the clinical trial as original research paper both in an adequate medical journal and to present the results at a congress. Marc Kriege is the first author / in the publication and the results presenting the prestigious conventions. Rüdiger Noppens, MD PhD select the journal with agreement of the co-authors.

**10. Signatures**

This study protocol was subjected to a critical review and was approved in the current version and wrote under - by those responsible. The information contained in it comply with:

The current benefit-risk assessment of study treatment. The moral, ethical and scientific principles of clinical research as in the Declaration of Helsinki and demonstrated to the GCP principles

**Principal Investigator**

Name: Marc Kriege, MD

| _________________ ____________________________________ |
| --- |
| Date Signature |
| **Study Nurse**  Name: Susanne Mauff   \| _________________ _____________________________________ \| \| --- \| \| Date Signature \| |

**11. Declaration of the Principal Investigator**

I have read the present study protocol and confirm that it contains all the information necessary to conduct the clinical trial. I agree that clinical trial as outlined in the study protocol perform.

I will not take the first patient in the clinical trial before all ethical and regulatory requirements are met. I declare that all participating in this clinical trial patients in the study protocol as required prior to the first study measure, obtain a written consent to participate in the clinical trial.

I am aware of the requirements of the correct reporting at all serious adverse events, and will the events as required by the study protocol, document and report.

I volunteer to make all study-related documentation and source data as described in the study protocol available. I will submit a resume before the clinical trial.

I will carry out the clinical trial in accordance with the study protocol, GCP and the required regulatory requirements.

**Principal Investigator**

Marc Kriege, MD

Langenbeckstrasse 1, 55131 Mainz

Phone: +49 (0) 6131 17- 0

Fax: +49 (0) 6131 17- 6902

E-Mail: makriege@uni-mainz.de

| _________________ _____________________________________ |
| --- |

Datum Unterschrift

**12. References**

1. Cooper RM, Pacey JA, Bishop MJ, McCluskey SA. Early clinical experience with a new

videolaryngoscope (GlideScope) in 728 patients. Can J Anaesth. 2005;52:191–198

2. Sun DA, Warriner CB, Parsons DG, Klein R, Umedaly HS, Moult M. The GlideScope Video Laryngoscope: randomized clinical trial in 200 patients. Br J Anaesth.

2005;94:381–384

3. Akihisa Y., Maruyama K., Koyama Y., Yamada R., Ogura A., Andoh T. Comparison of intubation performance between the King Vision and Macintosh laryngoscopes in novice personnel: a randomized, crossover manikin study. J Anesth.2014;28:51–57

4. Noppens, R.R., C. Werner, and T. Piepho, Indirekte Laryngoskopie: Alternativen zur Atemwegssicherung. Anaesthesist, 2010; 59: 149-161.

5. [Ludwig AA](http://www.ncbi.nlm.nih.gov/pubmed?term=Ludwig%20AA%5BAuthor%5D&cauthor=true&cauthor_uid=21544022), [Baulig W](http://www.ncbi.nlm.nih.gov/pubmed?term=Baulig%20W%5BAuthor%5D&cauthor=true&cauthor_uid=21544022), [Biro P](http://www.ncbi.nlm.nih.gov/pubmed?term=Biro%20P%5BAuthor%5D&cauthor=true&cauthor_uid=21544022).[A simulated severe difficult airway does not alter the intubation performance with the SensaScope: a prospective randomised manikin study.](http://www.ncbi.nlm.nih.gov/pubmed/21544022) Eur J Anaesthesiol. 2011; 28:449-453.

6. Savoldelli, G.L., et al. Comparison of the GlideScope^®^, the McGrath^®^, the Airtraq^®^ and the Macintosh laryngoscopes in simulated difficult airways*. Anaesthesia 2008; 63: 1358-1364.

7. Murphy L., Kovacs G., Reardon P., BSC , Law A. Comparison of the King Vision Video Laryngoscope with the Macintosh Laryngoscope. The Journal of Emergency Medicine. 2014; 47:239-246
